# Current Nanoparticle-Based Technologies for Osteoarthritis Therapy

**DOI:** 10.3390/nano10122368

**Published:** 2020-11-28

**Authors:** Guang-Zhen Jin

**Affiliations:** 1 Institute of Tissue Regeneration Engineering (ITREN), Dankook University, Cheonan 31116, Korea; gzhjin2002@dankook.ac.kr; Tel.: +82-41-550-1916; Fax: +82-41-559-7839; 2 Department of Nanobiomedical Science and BK21 PLUS NBM Global Research Center for Regenerative Medicine, Dankook University, Cheonan 31116, Korea; 3 Department of Biomaterials Science, College of Dentistry, Dankook University, Cheonan 31116, Korea; 4 Cell & Matter Institute, Dankook University, Cheonan 31116, Korea

**Keywords:** nanotechnology, nanoparticle, intra-articular delivery, osteoarthritis

## Abstract

Osteoarthritis (OA) is a common chronic joint disease that is characterized by joint pain and stiffness, and limitation of motion and the major cause of disability, which reduces life quality of patients and brings a large economic burden to the family and society. Current clinical treatment is mostly limited to symptomatic treatment aimed at pain alleviation and functional improvement, rather than suppressing the progression of OA. Nanotechnology is a promising strategy for the treatment of OA. In this review, we summarize the current experimental progress that focuses on technologies such as liposomes, micelles, dendrimers, polymeric nanoparticles (PNPs), exosomes, and inorganic nanoparticles (NPs) for their potential treatment of OA.

## 1. Introduction

Osteoarthritis (OA) is a common chronic joint disease that is characterized by joint pain and stiffness, and limitation of motion and the major cause of disability for adults [1]. The incidence of OA is related to age, gender, obesity, and joint injury etc., [2]. With its high prevalence, OA causes a significant financial burden both on individuals and the society [1]. OA pathophysiology is characterized by progressive and degenerative loss of articular cartilage, osteophyte formation, synovial inflammation, subchondral bone remodeling, and sclerosis. This disease is regarded as a complex disease that involves multiple tissues and processes. The causes are not yet fully understood [3]. In modern concepts, a variety of factors are associated with OA, including genetic susceptibility, biochemics, and biomechanics of the affected joint, and extent of inflammation [4]. Therefore, it has been difficult to identify specific targets for therapy. Current clinical practice is mostly limited on symptomatic treatment aimed at pain alleviation, functional improvement, and even artificial joint replacement, rather than targeting the underlying molecular causes of OA [5].

Articular cartilage is an elastic connective tissue covering the ends of the bones and helps joints to move smoothly. It lacks a vasculature supply and innervation, composed of 1–2% chondroocytes and specialized matrix involving type II collagen, glycosaminoglycans (GAGs, e.g., chondroitin sulfate, hyaluronic acid (HA), and aggrecan), elastin fibrils, and 70% water. The chondrocytes coordinate the synthesis, maintenance, and degradation of extracellular matrix (ECM) through the turnover of matrix proteins [6,7]. A joint cavity is filled with synovial fluid, which is surrounded by articular cartilage and a synovial membrane. The synovial membrane is composed of fibroblasts and macrophages and secrete two important molecules, namely hyaluronan and lubricin. They contribute to the viscosity of synovial fluid and provide boundary lubrication of articular cartilage [8].

**Figure 1:**
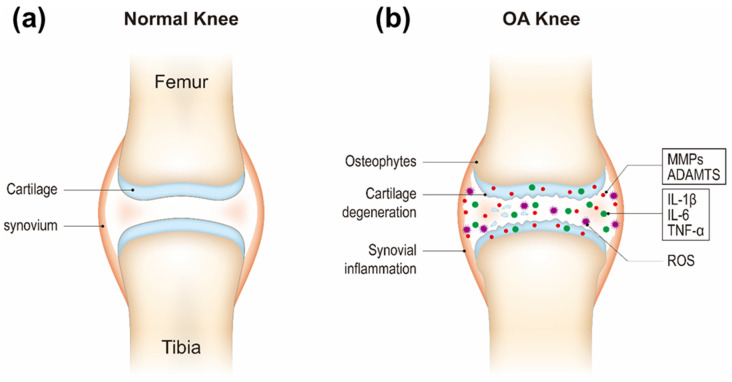
Schematic of healthy (**a**) and osteoarthritis (OA) knee joints (**b**).

An improved understanding of the pathogenesis of OA is very important for providing new theoretical basis and potential targets of OA clinical practice. The three major biological factors including proteolytic enzymes, proinflammatory cytokines, and reactive oxygen species (ROS) cause and exacerbate cartilage degradation in OA. Cartilage homeostasis relies on a balance between chondrocyte anabolic and catabolic activities. It is postulated that there is an imbalance between chondrocyte anabolism by growth factors and catabolism by decomposing enzymes such as matrix metalloproteinases (MMPs, e.g., MMP-3 and MMP-13) and a disintegrin and metalloproteinase with thrombospondin motifs (ADAMTS) [9]. These enzymes are generated by chondrocytes. ADAMTS regulate proteoglycan (e.g., aggrecan) degradation and MMPs digest the collagen network (e.g., type II collagen) [10,11]. The decomposition products contribute to further inflammatory responses of the adjacent synovial tissue through Toll-like receptors and integrins and the release of proinflammatory products including cytokines and ROS and lead to a defective cycle between the production of inflammatory factors and the chondrocyte catabolic activity [12,13,14]. Inflammatory cytokines (e.g., IL-1β, IL-6, and TNF-α) secreted by chondrocytes and synovial cells in synovial fluid are critical mediators in OA development [15]. They activate canonical nuclear factor kappa B (NF-κB) signaling, the major pathway mediating the expression of proinflammatory cytokines, involving IL-1β, IL-6, TNF-α, inducible nitric oxide synthase (iNOS), cyclooxygenase 2 (COX-2), and proteolytic enzymes (e.g., MMPs, ADAMTS) [16,17]. Therefore, NF-κB signaling pathway has become one of the potential targets for OA treatment. Recent studies have shown that OA progression is closely related to oxidative stress, which refers to increased levels of intracellular ROS produced from mitochondria and endoplasmic reticulum, resulting in lipids, proteins, and DNA damage [18,19,20]. Cartilage mechanical injury may result in the enhanced release of mitochondrial ROS [21]. ROS can up-regulate proinflammatory cytokine expression in OA [22,23], and the cytokines also induce ROS production [24], thereby accelerating OA development (Figure 1).

At present, pharmacological treatments including non-steroidal anti-inflammatory drugs (NSAID), opioids, and glucocorticoids are still the primary approaches. However, systemic administration of drugs can cause severe side effects for long-term use, such as gastrointestinal complications and osteoporosis [25,26]. In addition, the absence of blood supply and the rapid clearance of drugs within synovial joints are main challenges for systemical and local drug delivery, respectively [27]. Therefore, it is necessary to develop new drugs and new drug delivery systems for enhancing the therapeutic efficacy of OA.

## 2. Nanotechnology for OA Therapy

Nanotechnology is an interdisciplinary discipline including physics, chemistry, biology, electronics, and engineering. Research and development of nanotechnology are actively performed all over the world. Nanotechnology is a field of science for studying and manipulating particles at the atomic, molecular, or macromolecular levels, usually between 1 and 100 nm in size [28]. Due to the particularity of the scale structure, nanoparticles (NPs) have unique properties, including size effects, interfacial phenomena, and quantum effects, etc., thus exhibiting many excellent properties and new functions [28]. The behavior of NPs is more difficult to predict completely than that of microparticles. Therefore, the control and manipulation of nanostructures can eventually exploit novel chemical, physical, and biological characteristics of NPs. Nanotechnology has become very important because of its high ratio of surface area to volume, ideal scale for catalysis, and molecular structures at the nanoscale in the body [28]. Fabrication methods of NPs by nanotechnology usually include top-down approach and bottom-up approach. The top-down approach corresponds to using nanofabrication tools to create nanoscale particles through reducing macro-sized structures. On the other hand, the bottom-up approach utilizes physical and chemical processes to integrate molecular or atomic components into bigger nanoscale particles [29].

Nanotechnology has shown excellent application value in many aspects of our daily lives including sunscreens, cosmetics, textiles, and sports equipment. Nanotechnology is also used in biomedicine, some of which have entered clinical applications including Doxil^®^ for treating ovarian cancer, Ferumoxytol^®^ for treating of iron deficiency anemia, Abraxane^®^ for treating metastatic breast cancer, etc., [30]. However, there is still no current clinical application of nanotechnology for the treatment of OA.

Nanotechnology plays a unique advantage for drug delivery of therapeutics for OA: (1) Improving drug targeting and efficient drug delivery; (2) enhancing drug solubility and stability; (3) preventing drug dispersion and degradation in body fluids and extending drug circulation and retention time in the body; (4) improving drug efficacy and reducing adverse drug reactions [31].

**Figure 2:**
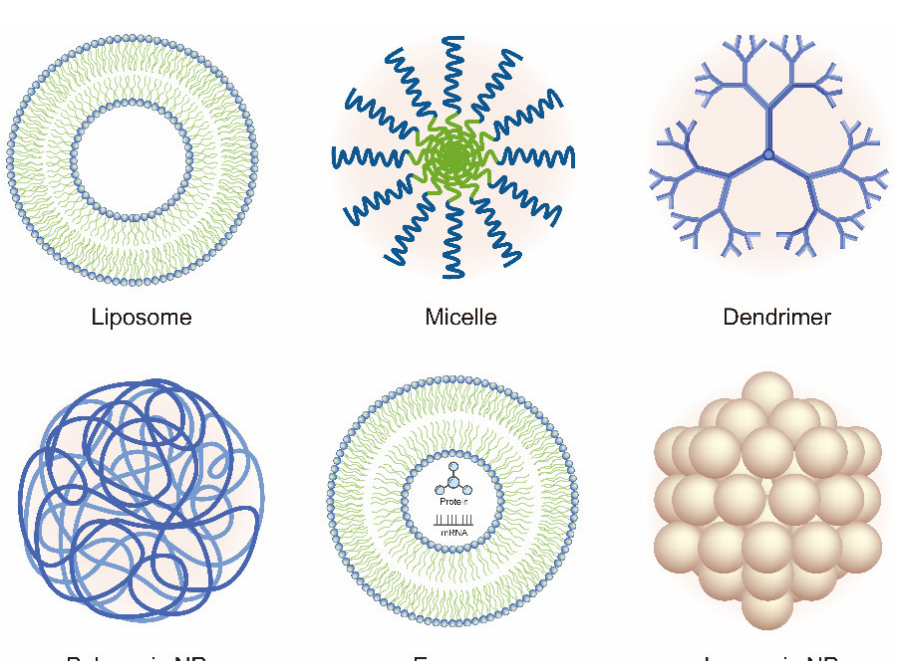
Schematic of the various nanoparticles (NPs) used in the treatment of OA.

**Figure 3:**
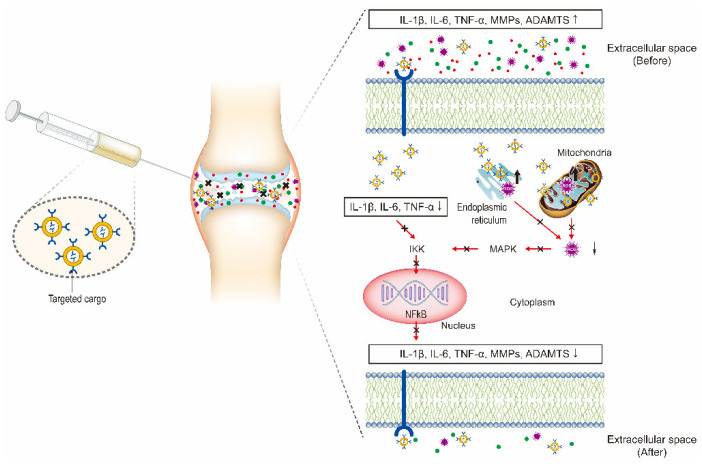
Intra-articular (IA) delivery of cargo-loaded nanoparticles for the treatment of OA. The therapeutic effects are obtained by the NPs targeting the three major factors including inflammatory factors, proteolytic enzymes, and reactive oxygen species (ROS) to inhibit NF-κB signaling pathway.

In recent years, the vigorous development of nanotechnology in drug delivery systems has provided new ideas and methods for OA therapy. In this review, we discuss the current developments and novel applications of OA-related NP-based drug delivery including liposomes, micelles, dendrimers, polymeric nanoparticles (PNPs), exosomes, and inorganic NPs. The application of NPs in the treatment of OA is summarized in Table 1 and Table 2. Various NPs used in the treatment of OA are shown in Figure 2. The delivery route and the schematic mechanism of NPs are displayed in Figure 3.

### 2.1. Liposomes

A liposome is an aqueous-core spherical vesicle surrounded by a phospholipid bilayer. The liposome size can vary from 50 to 5000 nm depending on the buffer and lipid composition. Morphologically, liposomes include small unilamellar vesicles, large unilamellar vesicles, and multilamellar vesicles. Small unilamellar vesicles are made of a single bilayer and are around 100 nm in size, large unilamellar vesicles with a size range of 200 to 800 nm and multilamellar vesicles with a size range of 500 to 5000 nm. Liposomal properties are highly manoeuvrable by modifying surface chemistry or coating polymers or attaching antibodies to create immunoliposomes [62].

Liposomes are considered to be the most ideal drug-delivery system and the first nano-drug carrier approved by FDA [63]. Several liposomal formulations have been used in clinical practice, e.g., AmBisome^®^ for anti-fungal [64], Doxil^®^ for anti-cancer [65], LiprostinTM for anti-thrombosis [66], etc. In particular, Lipotalon^®^ (dexamethasone palmitate) is used exclusively in the clinical treatment of OA by intra-articular (IA) delivery only in Germany [67]. The liposomal formulations are highly used in OA as drug-delivery systems not only because of their ability to encapsulate both hydrophilic and hydrophobic drug cargos within the phospholipid bilayer and the aqueous-core but also because of good safety profiles.

Adenosine is a critical autocrine factor for the maintenance of cartilage homeostasis. A2A receptor is one of several receptor subtypes for adenosine [68]. Corciulo et al. first encapsulated both adenosine and A2A receptor agonist in liposomes, then IA injections of cargos-loaded liposomes prevented OA progression in both obesity-induced OA in mice and post-traumatic OA in rats [32]. 

The results suggest that the A2A receptor is an effective target for OA treatment. Rapamycin is a specific inhibitor of mammalian target of rapamycin (mTOR). The mTOR is a potential therapeutic target of OA. The rapamycin exerts the potential therapeutic effects by PI3K/Akt/mTOR signaling cascade [69]. A recent study by Chen et al. showed that IA delivery of liposome-encapsulated rapamycin has significant anti-inflammatory effect in the spontaneous OA guinea [33]. Gold nanoparticles (GNPs) possess anti-arthritis activity due to their antioxidant and anti-inflammatory properties [70,71]. Sarkar et al. tagged GNPs with fish oil protein (FP) that has anti-inflammatory effects, then the FP-GNPs were encapsulated in dipalmitoyl phosphatidylcholine (DPPC) liposomes. The FP-GNP-DPPC was delivered by IA injection in rat models of OA. The results showed that FP-GNP was continuously released into the synovial fluid, both apoptotic markers such as Bax, Caspase 3, p53, etc., and pro-inflammatory cytokines such as TNF-a, IL-6, NF-kB, etc., were decreased as compared with the OA control group, and antioxidant markers such as glutathione reductase (GSH), superoxide dismutase (SOD), catalase, etc., were significantly improved than those of the OA control group. Therefore, the author suggested that FP-GNP-DPPC could be a novel anti-OA nano-drug [34]. Obesity is one of the pathophysiologic mechanisms of OA and increases M1 macrophage infiltration in the joint synovium [72]. Because clodronate liposomes cause macrophage depletion [73], Bader et al. recently demonstrated that IA delivery of clodronate-loaded liposomes reduced synovitis and cartilage degradation in mouse models of obesity-associated OA through macrophage depletion and collagen X reduction [35]. Thereby, their data provide good evidence for the potential targeting of macrophages in OA treatment. Curcumin is a yellow substance that has anti-inflammatory and antioxidant activities. It is commonly used for arthritis, respiratory infections, and cancer [74]. Because curcumin has very low bioavailability, Yeh et al. encapsulated curcumin in soybean phosphatidylcholines liposomes to increase the bioavailability of IA delivery. The liposome formulation could increase cellular uptake of curcumin and downregulated the expression of inflammatory markers in an in vitro OA model established by interleukin-1β [75]. Therefore, curcumin-loaded soybean phosphatidylcholines liposomes may slow OA progression. Liposomal formulations have various advantages such as excellent biocompatibility, low toxicity, and entrapping both lipophilic and hydrophilic drugs. However, liposomes also have some disadvantages such as leakage of encapsulated drug, physical instability, and rapid clearance from the synovial fluid. Therefore, these drawbacks still remain a challenge for liposome-based IA drug-delivery system.

### 2.2. Micelles

Micelles are nanoscale amphiphilic structures that have a hydrophobic core and a hydrophilic shell with the size ranging from 5 to 100 nm [76]. The size of the micelle varies depending on the properties of the amphiphile and drug entrapment. The micelle can carry hydrophobic drugs within its core while its shell binds hydrophilic drugs. A critical micelle concentration (CMC), a key parameter for micelles, is the minimal amphiphile concentration for micelle formation. The self-assembled structures occur as soon as the concentration of amphiphile in the aqueous solution reaches the CMC [77]. Micelles can also be preferentially uptaken by conjugating with peptides, antibodies, or other targeting ligands [78]. Among the different kinds of micelles, polymeric micelles are most widely used in drug delivery systems [79]. They are made up of block-copolymers consisting of hydrophilic and hydrophobic chains. Due to the low CMC, polymeric micelles remain more stable and possess a longer circulation time when compared with other micelles [80].

The polymeric micelles are mainly used in cancer clinical practice, and rarely explored for OA therapy [81]. During OA inflammation, synovial fluid is acidic and overexpressed MMP-13 [82,83]. Psoralidin (PSO), a traditional Chinese medicine, has anti-inflammatory effects on OA [84,85]. Poly (2-ethyl-2-oxazoline)-poly (ε-caprolactone) (PPL) is an acidic pH-responsive polymer. Lan et al. first grafted a specific collagen type II targeting peptide (Coll-II α1 chain-binding peptide–CollB) onto PPL (C-PPL). In parallel, a specific peptide substrate of MMP-13 enzyme was conjugated onto PPL (MR-PPL). Lastly, psoralidin was loaded into the theranostic nanoplatform self-assembled by C-PPL and MR-PPL (MRC-PPL) [36]. Then they delivered psoralidin-loaded MRC-PPL into the joint cavity of mouse models of papain-induced OA. After 6 weeks post-treatment, the cartilage lesions were significantly alleviated by down-regulating MMP-13 and the anti-OA effects were exerted by NF-κB signaling pathway. Poly (β-amino ester) (PAE) is a cationic polymer with low cytotoxicity [86]. pH can adjust hydrophobic block/hydrophilic block transition of PAE by ionization/deionization transition of tertiary amine [87]. The positive charges of PAE are beneficial for targeted drug delivery by electrostatic interactions with GAGs of cartilage [88]. Kang et al. used the two properties of PAE to design acid-activatable curcumin polymer (ACP), which was covalently incorporated with curcumin. The therapeutic effects of ACP micelles were evaluated in mouse models of monoidoacetic acid (MIA)-induced OA. The results showed that ACP micelles significantly protected the articular cartilage of OA by down-regulating TNF-α and IL-1β. The author rationally used the pathological microenvironment of OA to design the logical strategy for OA treatment. Thereby, they also provide useful information on the methodology for the treatment of OA [37]. Kartogenin (KGN) is a compound screened from 22,000 heterocyclic molecules [89]. It can promote chondrogenic differentiation of human mesenchymal stem cells (MSCs) by up-regulating the chondrogenic genes. Kang et al. first prepared self-assembled PEGylated kartogenin (PEG/KGN) micelles by covalent crosslinking between polyethylene glycol (PEG) and KGN, then obtained HA/PEG/KGN hydrogels by covalently bonding PEG chains to HA. The in vitro release study showed that KGN from HA/PEG/KGN hydrogels was significantly delayed than that of PEG/KGN micelles with time. The in vivo study revealed that IA injections of HA/PEG/KGN hydrogels significantly suppressed the progression of OA in rat surgically induced models when compared with control groups. The results suggest that the HA/PEG/KGN hydrogels provide new prescription for the treatment of OA [38]. Micelles have some advantages such as improving solubility of highly lipophilic drugs, tunable chemical and physical properties, and drug release in controlled manner. However, they have certain shortcomings such as non-encapsulating hydrophilic drugs, CMC dependency, and their toxicity concerns. To overcome these limitations, modification of micelles might prove to be necessary.

### 2.3. Dendrimers

Dendrimers are repetitively branched macromolecules with tree-like topological nanostructures. The dendrimers consist of three different components: the core, the branches, and the shell. The shell provides outer surface of the dendrimers that can be used for conjugation with cargo or targeting ligands. The hydrophobic core can carry the hydrophobic cargo. The size of the dendrimers is determined by the number of generations present in their structure [90]. Dendrimers, as a drug delivery system, possess advantages such as well-defined number of surface functional groups, monodispersity, controllable size, and high efficiency of cargo payload [91,92]. Polyamidoamine (PAMAM) dendrimers and polypropylene imine dendrimers are the two most commonly used dendrimers.

There are many preclinical literatures about the application of dendrimers. VivaGel^TM^ is a first dendrimer-based commercial medical product for the prevention of HIV and HSV infection [93]. Other products from dendrimers used in clinical trials include the following: ImDendrim for liver cancer, DEP^®^ docetaxel and DEP^®^ cabazitaxel for breast cancer, and OP-101 for X-linked adrenoleukodystrophy [94]. The routes of administration of dendrimers as drug carriers include cutaneous, intravenous, oral, etc., [95] However, dendrimers are rarely studied in the treatment of OA in recent years. The degeneration of OA cartilage is due to catabolism exceeding anabolism in chondrocytes. Insulin-like growth factor 1 (IGF-1) is an anabolic growth factor that promotes the biosynthesis of cartilage matrix and shows anti-inflammatory effects [96]. Geiger et al. targeted the anionic cartilage tissue of OA by designing cationic PEGylated PAMAM which was conjugated with IGF-1 [39]. The in vivo study showed that PEGylated dendrimer–IGF-1 penetrated efficiently into the full thickness of rat articular cartilage via electrostatic interactions and rescued cartilage degeneration in rat models of surgically induced OA. These results of the study provide useful information for clinical trials of new drug treatment of OA. As mentioned above, partly PEGylated PAMAM dendrimers were used as the carrier of anti-OA drug. Interestingly, Hu et al. from another research group conjugated KGN to the surface of PAMAM and the end group of PEG to obtain PEG-PAMAM-KGN (PPK) and KGN-PEG-PAMAM (KPP) conjugates, respectively. However, PPK and KPP have different effects on the in vitro chondrogenic differentiation of MSCs. The data showed that KPP promoted higher chondrogenic differentiation of MSCs than PPK. Therefore, the authors suggest that PEG-PAMAM could be a useful nano-drug carrier system for OA treatment [97]. Dendritic polyglycerol sulfate (dPGS) is another type of dendrimer consisting of glycerol units and sulfate groups. It can reduce the levels of complement C3 and C5 and the extravasation of white blood cells. So dPGS has anti-inflammatory activity [98]. In rat models of surgically induced OA, dPGS was administered subcutaneously once daily for 2 weeks. After 8 weeks’ post-treatment, dPGS decreased the Mankin and Glasson score values when compared with control groups. Therefore, dPGS can suppress OA progression through a chondroprotective and anti-inflammatory effects [40]. Dendrimers possess various advantages such as increasing solubility of hydrophobic drugs and tunable physicochemical properties and some disadvantages such as non-entrapping hydrophilic drugs and cellular toxicity similar to that of micelles. In particular, a unique property of dendrimers is multiple functional groups in their structure. Therefore, dendrimers are considered as potential carriers for targeted drug delivery. Their cellular toxicity can be modulated by surface moieties.

### 2.4. PNPs

PNPs are defined as solid particles with size range from 10–1000 nm [99]. PNPs are composed of biocompatible and biodegradable synthetic polymers such as poly(lactide) (PLA), poly(lactide-co-glycolide) copolymers (PLGA), poly (ε-caprolactone) (PCL), etc., and natural polymers such as chitosan, alginate, gelatin, albumin, and so on [100]. PNPs have two types of structural forms: nanospheres and nanocapsules. Nanospheres consist of polymer matrix on which the drug is uniformly dispersed whereas nanocapsules are nanostructures with a reservoir core in which the drug is surrounded by a polymeric membrane [101].

The synthesis of PNPs is relatively facile as compared to that of other NPs, so they are widely used in the field of nanomedicine. The functions of PNPs for nano-drug delivery usually include the following: (1) extending drug half-life, and (2) controlling drug release. Many different types of PNPs have been used in clinical trials, e.g., Copaxone^®^ for multiple sclerosis [102], Neulasta^®^ for neutropenia [103], Abraxane^®^ for breast cancer [104], etc. In particular, Abraxane^®^ is a conjugate of natural albumin drug approved by the FDA in 2005. So far, there is no report that PNPs as drug carriers are used in OA clinical trials. Mitochondria are the main site of ROS generation, and p66shc (an isoform of the shcA adaptor protein family) and p47phox (an NADPH oxidase subunit) play a crucial role in ROS production [105,106,107]. Shin et al. first respectively encapsulated p66shc siRNA and p47phox siRNA in PLGA by an emulsification/solvent evaporation method to obtain p66shc si-PLGA and p47phox si-PLGA NPs. Then both the NPs were respectively administered by IA injection in rat models of MIA-induced OA. The results showed that both the NPs ameliorated cartilage disruption by reducing inflammatory cytokine and ROS production [41,42]. Therefore, IA delivery of siRNA using PLGA NPs as a drug carrier may represent a promising novel strategy for the OA treatment. Pain relief using NSAIDs is also a traditional pharmacological therapy of OA. Etoricoxib is a COX-2 selective NSAID approved for clinical practice in 2002. It reduces the transformation of arachidonic acid to prostaglandin E_2_ by inhibiting COX-2 [108]. Because it has adverse cardiovascular effects when administered systemically [109], the local route of etoricoxib administration, such as direct IA injection, should be considered. Liu et al. used PLGA-PEG-PLGA triblock copolymeric NPs as a drug-delivery system to load etoricoxib using oil in water emulsion solvent evaporation method. They then administered the etoricoxib-loaded NPs by IA injection in rat models of surgically induced OA. The data showed that the etoricoxib-loaded NPs alleviated the signs and symptoms of OA through down-regulating the expressions of COX-2, prostaglandin E2, iNOS, MMP-13, and ADAMTS-5 [43]. Conjugation of pharmacologically active small molecules and less degradable NPs is an effective approach to increase the half-life of the molecules. As mentioned above, adenosine plays a very important role in maintaining cartilage homeostasis. However, it has an extremely short half-life in body fluids. Liu et al. first synthesized PLA-PEG block copolymer NPs, then were conjugated to adenosine using click chemistry reaction to obtain adenosine-functionalized NPs. For in vivo animal study, the adenosine NPs were injected into the joint cavities of rat models of post-traumatic OA. The results revealed that the NPs prevented OA progression via NF-κB signaling pathway. Finally, the authors suggest that attachment of adenosine to PLA-PEG block copolymer NPs will provide a novel approach for extending the therapeutic efficacy of OA [44]. Polyurethanes are important biomedical polymers that carry urethane bonds in their main chains. They were first used commercially for biomedical application in 1960 [110]. Fan et al. first synthesized amphiphilic polyurethane NPs with pendant amino group, then prepared polyurethane-KGN conjugate by covalently bonding between the amine group of polyurethane and the carboxyl group of KGN. The therapeutic results in rat OA models showed that IA injection of polyurethane-KGN NPs could retain more cartilage matrix and suppress the development of OA [45]. Nanocrystal technology is a promising technology for the delivery of poorly soluble drug with size range from 100 to 1000 nm, such as KGN [111]. Polymeric particles with a size of 10 to 25 µm possess longer retention times in joint cavities following IA delivery [112]. Maudens et al. embedded KGN NPs with 320 nm in PLA microparticles with a mean size of 13.81 µm using both the techniques. The in vitro drug release analysis showed that KGN-PLA particles had an extended drug release of 62% over 90 days. The in vivo study revealed that KGN-PLA particles protected osteochondral lesions from mouse OA models. This work provides a good example of extending drug retention for the treatment strategy of OA [46]. HA is the main component of articular cartilage and synovial fluid. Due to the degradation of endogenous HA during OA progression, IA injection of exogenous HA plays an important role in the treatment of OA by its lubricating and anti-inflammatory effects [113]. A study showed the reason why HA cannot effectively exert its physiological effects including its faster degradation and inability to localize on the cartilage surface [114]. Collagen-binding peptide (COLBP) possesses a property that can localize to the cartilage surface [115]. Therefore, Faust et al. used a peptide-polymer platform to bind HA-binding peptide (HABP) to PEG-COLBP conjugate. The in vitro analysis using quartz crystal microbalance and isothermal calorimetry revealed that the peptide-polymers had a high affinity to HA. The peptide-polymers were delivered to the joint cavities of young and aged mouse OA models. The results showed that the peptide-polymers were well localized to both cartilage defects and synovium, and similarly suppressed the cartilage degeneration of both the mouse models [47]. Chitosan, a linear polysaccharide, is one of the most successfully developed natural polymers because of its biosafety, non-toxicity, low immunogenicity, biocompatibility, and biodegradability [116]. Chitosan nanoparticles (CNPs) have been widely recognized as a drug-delivery carrier. They have many different preparation techniques including ionic emulsion cross-linking, spray-drying, desolvation with cationic salts, ionic gelation, etc. [117]. CNPs are combined with berberine chloride (BBR), which is a natural insoluble compound with anti-inflammatory effects, by the ionic cross-linking method. BBR-CNPs exerted anti-OA efficacy through the extended release of BBR in rat models [48]. In another study, curcumin was loaded to HA/CNPs by the ionic cross-linking method, then curcumin-loaded HA/CNPs were delivered into the joint cavities of rat OA models by IA injection. The results showed that HA and curcumin suppressed synergistically the development of OA by down-regulating NF-κB and MMP-13 and up-regulating type II collagen [49]. IL-1β is one of the main therapeutic targets of OA, so the inhibition of IL-1β generation is a feasible treatment strategy. Cytokine response modifier A (CrmA) is a potent inhibitor of interleukin-1β converting enzyme [118]. Zhou et al. first synthesized plasmid DNA-CrmA, then CrmA-HA-CNPs were prepared by the complex coacervation of cationic polymers. Thereafter, the anti-OA effects of the NPs were analyzed in rat surgically induced models. After 12 weeks’ post-operation, the NPs significantly attenuated cartilage destruction by inhibiting IL-1β formation [50]. PNPs offer several advantages such as incorporation of hydrophilic and hydrophobic drugs, controlled drug release, and higher stability. On the other hand, they have also some drawbacks such as poor drug loading and toxicity concerns. In order to overcome the problems, various modifications and strengthening toxicology research are still required.

### 2.5. Exosomes

Exosomes are endosome-derived membrane-bound phospholipid bilayer vesicles with diameters of 50–150 nm. The cargo of exosomes includes nucleic acids (DNAs, mRNAs, microRNAs, and IncRNA), bioactive lipids, and proteins that can be transferred between cells [119]. Exosomes can be secreted by almost all cell types of normal and pathological cells. They are presented in body fluids in vivo including blood, urine, saliva, breast milk, and synovial fluids and the conditioned medium of all types of cells in vitro [120,121].

Many studies have confirmed that MSCs have potential therapeutic effects for OA [122,123,124,125,126]. The effects are mainly attributed to MSC-secreted secretome and exosome [127,128,129,130]. Secretome of MSCs include all the proteins secreted by the MSCs, including growth factors, cytokines, hormones, etc., [131]. As mentioned earlier, however, exosomes are mainly composed of nucleic acids including mRNAs, microRNAs (miRNAs), and IncRNA. Moreover, exosomes used in treating OA are principally derived from MSCs, as shown in the Table 2. Recent studies have shown that exosomal miRNAs [51,52,53,54,55,132,133] and IncRNAs [56,57,134,135] play a critical role in the anti-OA efficacy. Although the content of exosomes was not mentioned in several studies of this review, the therapeutic effects of OA are definite [58,59,60,61]. For instance, as stated above, drug delivery through the dense cartilage matrix is still a major challenge. To overcome this obstacle, Liang et al. fused a chondrocyte-affinity peptide (CAP) on the surface of exosomes derived from chondrocytes using the lysosome-associated membrane glycoprotein 2b. The in vivo study showed that CAP-exosome-based miR-140 delivery via IA injection significantly alleviated the development of OA in rat models [51]. Anti-OA mechanisms of MSC-derived exosomes are currently being explored. Several recent studies indicate that miRNAs may regulate the expression of genes involved in catabolism and anabolism of OA at upstream levels of several signaling pathways, such as NF-kB pathway, Wnt/β-Catenin pathway, and SIRT1/p53 pathway [136,137,138,139,140]. Therefore, the exosomes could inhibit the production of pro-inflammatory cytokines (e.g., IL-1β, IL-6, TNF-α) and proteolytic enzymes (e.g., MMPs, ADAMTS) by these signaling pathways. Naturally occurring exosomes not only possess therapeutic cargo themselves, but also they can serve as carriers for drug delivery [141,142,143]. However, the small amounts of exosomes released from MSCs are not enough for clinical research. Therefore, it is currently a technical challenge to obtain a sufficient amount of exosomes for in vivo use. To improve exosomes yield, some studies are exploring and using hypoxic three-dimensional spheroid culture, microvesicles, and cellular-nanoporation methods [144,145,146]. With the elucidation of the mechanism of action and the maturity of manufacturing technologies of exosomes, we believe that it will usher in a new era of OA treatment.

### 2.6. Inorganic NPs

There are three main antioxidant enzymes in the human body, namely peroxidase, catalase, and superoxide dismutase (SOD). They prevent the damage caused from oxidative stress by scavenging ROS [147]. However, the activity of these natural enzymes is easily lost because of the influence of the surrounding microenvironment such as pH, temperature, and proteases. Therefore, many studies are focused on the development of nanozymes. A nanozyme is a type of NPs with natural enzyme-like activity [148]. Among the numerous nanozymes, inorganic NPs including cerium oxide (CeO_2_), manganese dioxide (MnO_2_), platinum (Pt), etc., have attracted significant attention in the field of biomedicine because of their multi-enzymatic activity. For instance, CeO_2_ and Pt NPs mimic SOD-, catalase-, and peroxidase-like activities, and MnO_2_ NPs mimic SOD- and catalase-like activities [148]. They have been demonstrated as efficient antioxidants for cytoprotection [149,150,151,152,153,154].

**Figure 4:**
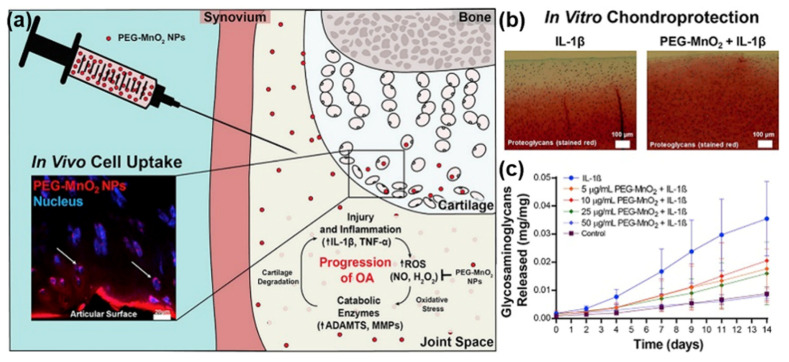
Anti-OA effects of MnO2 NPs. (**a**) The chondroprotective mechanism of MnO2 NPs and their intracellular localization in IL-1β-induced OA model. (**b**,**c**) MnO2 NPs preserved the ECM of cytokine-challenged explants. Reproduced from [157], with permission from Copyright Elsevier, 2019.

Lin et al. used cerium(III) nitrate hexahydrate and potassium carbonate to synthesize CeO_2_ NPs with a size of 120 nm by hydrothermal method. They confirmed that CeO_2_ NPs protected chondrocytes against damage induced with H_2_O_2_ through scavenging of ROS [155]. Ponnurangam et al. used a commercial CeO_2_ NPs with a size of 65 nm × 8 nm to treat the damaged chondrocytes induced by IL-1α in an in vitro model of chronic OA. The results showed that the CeO_2_ NPs also had an obvious anti-inflammatory effects on chronic chondrocyte inflammation [156]. To evaluate the anti-OA effects of MnO_2_ NPs, Kumar et al. first synthesized MnO_2_ NPs by the oxidation of potassium permanganate with poly (allylamine hydrochloride), then treated with MnO_2_ NPs in an ex vivo bovine model of IL-1β-induced chronic OA. The data revealed that the MnO_2_ NPs could prevent the development of OA by reducing ROS-induced oxidative stress (Figure 4) [157]. As mentioned above, numerous nanozymes have been confirmed for their cytoprotection, but traditional chemical and physical methods are harmful to environment and human beings. Therefore, green synthesis of nanozymes has attracted attention because it is environmentally friendly and minimizes adverse effects to human. The green synthesis is a new approach of synthesizing NPs using microbes and plant extracts [158,159]. Yin et al. used chloroplatinic acid and chondroitin sulfate by just heating to biosynthesize Pt NPs with a size range of 3 to 5 nm. The in vitro bioactivity analysis showed that the Pt NPs are biocompatible against human OA chondrocytes up to a concentration of 10 ppm. The results suggest the potential of Pt NPs for treating OA [160]. The main obstacle encountered by inorganic NPs is their toxicity concerns due to insufficient toxicological assessment in the literature. It is necessary to obtain reliable experimental data through strengthening toxicology research for OA treatment.

## 3. Challenge and Perspective in OA Therapy

Although the increasing advances on the understanding of the pathological mechanism of OA, the effective treatment of OA still faces great challenges. The current clinical treatment of OA is only for delaying the development of the disease, reducing pain and improving movement function, and there is no curable method in nature. As described in this review, NPs-based drug delivery systems show promise for the treatment of OA, including targeted drug biodistribution, extended drug release, and prolonged drug retention.

It is very important to choose which technique to establish OA animal models according to different research objectives. The models are usually established using three techniques including chemical, surgical, and naturally occurring methods. The OA models summarized in Table 1 and Table 2 are mainly surgery- and chemistry-induced models, only two cases are naturally occurring models. However, the first two models are not suitable for observing the effect of drugs in early OA because of the interference of synovitis caused by chemical and surgery. The naturally occurring model is not disturbed by traumatic synovitis and is closer to the pathological process of OA. Inappropriate choice of animal models is also an important point for delaying the development of effective therapies of OA. Consequently, the naturally occurring models are strongly recommended for use of IA drug delivery of OA.

As mentioned earlier, current researches provide opportunities for disease improvement by focusing on anabolic-catabolic balance strategies. In particular, the effect of KGN for cartilage repair by promoting chondrogenesis of endogenous MSCs was published in Science in 2012 [89]. Since then, numerous studies have shown similar findings. Several researches listed in this review have further confirmed the effectiveness of KGN in cartilage repair [38,46,97,111]. KGN not only promotes the chondrogenic differentiation of MSCs, but also improves the proliferation and survival of chondrocytes. Therefore, KGN is one of the best candidates in a number of therapeutic agents of OA. Nanozymes, such as CeO_2_, MnO_2_, and Pt NPs, mimic natural antioxidant enzymes and possess strong ROS-scavenging activities. The delivery of KGN by using nanozymes as carriers may be a promising strategy for OA treatment.

More and more studies demonstrate the effectiveness of miRNAs for the treatment of OA. As mentioned above, miRNAs have key roles in both cartilage development and homeostasis with age. Therefore, miRNAs is another excellent candidate in therapeutic agents of OA, although the mechanism of action of miRNAs in OA treatment remains to be further elucidated. We believe that the combination of the novel nanotechnology with miRNAs as cargo in appropriate OA models will achieve optimal therapeutic outcomes in the near future.

## Figures and Tables

**Table 1:** Intra-articular drug delivery nanoparticles for the treatment of OA.

Type of NPs	Formulation	OA Model/Route of Delivery	Outcome	Ref
**Liposomes**	Adenosine; CGS21680	Bbesity-induced (mice)/i.a. Post-traumatic (rats)/i.a.	Favorable histology and prevent OA progression	[32]
	Rapamycin	Spontaneous (guinea pigs)/i.a.	IL-6 ↓; MMP-13 ↓; collagen II ↑; OARSI score ↓; favorable histology	[33]
	FP/GNP/DPPC	Collagenase (rats)/i.a.	Anti-apoptosis ↑; pro-inflammatory cytokines ↓; GSH/ SOD/catalase ↑; NFκB ↓	[34]
	Clodronate	Post-traumatic (mice)/i.a.	M1 macrophages ↓; collagen X ↓; favorable histology	[35]
**Micelles**	MRC-PPL/Psoralidin	Papain (mice)/i.a.	MMP-13 ↓TNF-α ↓; NFκB ↓; favorable histology	[36]
	PAE/Curcumin	MIA (mice)/i.a.	IL-1β ↓; TNF-α ↓; favorable histology	[37]
	HA-PEG/KGN	Post-traumatic (rats)/i.a.	OARSI score ↓; favorable histology and prevent OA progression	[38]
**Dendrimers**	PAMAM/IGF-1	Post-traumatic (rats)/i.a.	Synovial inflammation scores ↓; area of degenerated cartilage ↓ favorable histology and μCT	[39]
	dPGS	Post-traumatic (rats)/s.c.	Mankin score ↓; Glasson score ↓; favorable histology	[40]
**PNPs**	p66shc si-PLGA	MIA (rats)/i.a.	IL-1β ↓; TNF-α ↓; COX2 ↓ favorable histology and μCT	[41]
	p47phox si-PLGA	MIA (rats)/i.a.	ROS ↓; favorable histology	[42]
	Etoricoxib/PLGA-PEG-PLGA	Post-traumatic (rats)/i.a.	COX2 ↓; iNOS ↓; MMP-13 ↓; ADAMTS-5 ↓; OARSI score ↓; favorable histology and μCT	[43]
	PLA-PEG-adenosine	Post-traumatic (rats)/i.a.	NFκB ↓; OARSI score ↓; favorable histology	[44]
	Polyurethane-KGN	Post-traumatic (rats)/i.a.	OARSI score ↓; favorable histology	[45]
	KGN-PLA	Post-traumatic (mice)/i.a.	OARSI score↓; favorable histology	[46]
	HABP-PEG-COLBP	Post-traumatic (mice)/i.a.	IL-1β ↓; IL-6 ↓; MMP-13 ↓; OARSI score↓; favorable histology	[47]
	BBR-CNPs	Post-traumatic (rats)/i.a.	Bcl-2 ↑; bax ↓; caspase-3 ↓; favorable histology	[48]
	Curcuminoid-HA-CNPs	Post-traumatic (rats)/i.a.	NFκB ↓; MMP-1 ↓; MMP-13 ↓; collagen II ↑; favorable histology	[49]
	CrmA-HA-CNP	Post-traumatic (rats)/i.a.	IL-1β ↓; MMP-3 ↓; MMP-13 ↓; OARSI score↓; favorable histology	[50]

Abbreviations: BBR: Berberine chloride; BDMC: bisdemethoxycurcumin; CNPs: Chitosan nanoparticles; COLBP: Collagen binding peptide; dPGS: Dendritic polyglycerol sulfates; DPPC: dipalmitoyl phosphatidylcholine; FP: Fish oil protein; GNPs: Gold nanoparticles; GSH: Glutathione reductase; HA: Hyaluronic acid; HABP: Hyaluronic acid-binding peptide; HA/CS-CrmA: Hyaluronic acid-chitosan nanoparticles containing plasmid DNA encoding CrmA; i.a.: intra-articular; IGF-1: Insulin-like growth factor 1; KGN: Kartogenin; MIA: Monoidoacetic acid; MRC: MMP-13 responsive/Coll-II α1 chain-binding peptide–CollB; OARSI: Osteoarthritis Research Society International; PAE: Poly(β-amino ester); PAMAM: polyamidoamine; PEG: poly (ethylene glycol); PNPs: Polymeric nanoparticles; PPL: Poly (2-ethyl-2-oxazoline)-poly (ε-caprolactone); s.c.: subcutaneous; SOD: Superoxide dismutase.

**Table 2:** The therapeutic effects of exosomes derived from different sources for OA.

Source	Cargo	OA Model/Route of Delivery	Outcome	Ref
Human chondrocytes	miR-140	Post-traumatic (rats)/i.a.	OARSI score ↓; favorable histology	[51]
Rat BMSCs	miR-9-5p	Post-traumatic (rats)/i.a.	IL-1 ↓; IL-6 ↓; TNF-α ↓; MMP-13 ↓; COMP ↓; SDC1 ↓; favorable histology	[52]
Human IPFP MSCs	miR-100-5p	Post-traumatic (mice)/i.a.	Collagen II ↑; MMP-13 ↓ ADAMTS-5 ↓; mTOR ↓; favorable histology	[53]
Rat BMSCs	miR-135b	Post-traumatic (rats)/i.a.	OARSI score ↓; Sp1 ↓	[54]
SM-MSCs	miR-140-5p	Post-traumatic (rats)/i.a.	RalA ↑; OARSI score ↓ favorable histology	[55]
Human BMSCs	lncRNA KLF3-AS1	Collagenase II (rats)/i.a.	MMP-13 ↓; Mankin score ↓; favorable histology	[56]
Human BMSCs	lncRNA-KLF3-AS1	Collagenase II (mice)/i.a.	MMP-13 ↓; GIT1 ↑	[57]
Rat BMSCs	Not mentioned	MIA (rats)/i.a.	IL-1β↓; IL-6↓; TNF-α↓; MMP-13↓; pain↓; favorable histology	[58]
Mouse BMSCs	Not mentioned	Collagenase VII (mice)/i.a.	TNF-α ↓; MMP-13 ↓; favorable histology and μCT	[59]
human ESC-MSCs	Not mentioned	Post-traumatic (mice)/i.a.	ADAMTS-5 ↓; OARSI score ↓; favorable histology	[60]
SM-MSCs; iPSC-MSCs	Not mentioned	Collagenase (mice)/i.a.	ICRS ↓; OARSI score ↓ favorable histology	[61]

Abbreviations: AFSC: Amniotic fluid stem cells; BMSCs: Bone marrow mesenchymal stem cells; ESC: Embryonic stem cell; GIT1: G-proteincoupled receptor kinase interacting protein-1; i.a.: intra-articular; ICRS: International Cartilage Research Society; IPFP: Infrapatellar fat pad; iPSCs: Induced pluripotent stem cells; MIA: Monoiodoacetate; mTOR: Mammalian target of rapamycin; SDC1: Syndecan-1; SM: Synovial membrane; Sp1: Specificity Protein 1; TGFβ: Transforming growth factor β.
